# IMU/Magnetometer-Based Azimuth Estimation with Norm Constraint Filtering

**DOI:** 10.3390/s24102982

**Published:** 2024-05-08

**Authors:** Chuang Yang, Qinghua Zeng, Zhi Xiong, Jinxian Yang

**Affiliations:** 1 College of Automation Engineering, Nanjing University of Aeronautics and Astronautics, Nanjing 211106, China; yangchuang@nuaa.edu.cn (C.Y.); xiongzhi@nuaa.edu.cn (Z.X.); 2 State Key Laboratory of Airliner Integration Technology and Flight Simulation, Nanjing 211106, China; 3 College of Electrical Engineering, Henan Polytechnic University, Jiaozuo 454000, China; yang_ch126@126.com

**Keywords:** measurement while drilling, azimuth estimation, rotary norm constraint filtering, geomagnetic field

## Abstract

A typical magnetometer-based measurement-while-drilling (MWD) system determines the azimuth of the bottom hole assembly during the drilling process by employing triaxial accelerometers and magnetometers. The geomagnetic azimuth solution is susceptible to magnetic interference, especially strong magnetic interference and so a rotary norm constraint filtering (RNCF) method for azimuth estimation, designed to support a gyroscope-aided magnetometer-based MWD system, is proposed. First, a new magnetic dynamical system, one whose output is observed by the magnetometers triad, is designed based on the Coriolis equation of the desired geomagnetic vector. Second, given that the norm of the non-interfered geomagnetic vector can be approximated as a constant during a short-term drilling process, a norm constraint procedure is introduced to the Kalman filter. This is achieved by the normalization of the geomagnetic part of the state vector of the dynamical system and is undertaken in order to obtain a precise geomagnetic component. Simulation and actual drilling experiments show that the proposed RNCF method can effectively improve the azimuth measurement precision with 98.5% over the typical geomagnetic solution and 37.1% over the KF in a RMSE sense when being strong magnetic interference environment.

## 1. Introduction

Due to GNSS service outages that occur underground, the oil drilling industry employs mainly magnetometer-based MWD systems to navigate the drill bit through an estimating of the orientation (including inclination and tool face angle, as well as azimuth) of the bottom hole assembly (BHA) during the drilling process [1,2,3]. By using accelerometer/magnetometer triads to measure the projections of the three-dimensional geographical field vectors (i.e., the Earth’s gravity field and magnetic field) on the BHA frame, a magnetometer-based MWD system can autonomously provide a real-time orientation estimation of the BHA, avoiding accumulative error in the gyroscope triad case. Such a system can deliver a high-precision inclination/tool face angle of the BHA because inclination/tool face angle-dependent components of the readings from the accelerometer triad, i.e., the Earth’s gravity vector, possesses a strong tolerance for external interference. Unfortunately, the magnetic interference from the drilling equipment (e.g., metallic materials, magnetic debris in the drilling fluid) and the randomly located ore deposits and other unknown interference sources may easily mask the desired azimuth-dependent part of the readings from the magnetometer triad, i.e., the geomagnetic vector, leading to serious distortion of the geomagnetic azimuth and even to a failure to drill to the destination [4,5]. Therefore, to improve azimuth precision, the magnetic readings from the magnetometer triad should be effectively preprocessed in advance.

To weaken magnetic interference from metallic drilling equipment, a magnetometer-based MWD system is typically mounted inside an expensive non-magnetic drill collar [6]. This can effectively remove magnetic interference, due to the metallic materials of the drill collar; however, use of a non-magnetic drill collar imposes the problem of high cost [7]. The removal of magnetic interference via software offers an alternative approach by which to cancel out the magnetic interference that derives from metallic materials and is achieved by characterizing mathematical models of these materials [8,9,10]. To alleviate magnetic interference due to magnetic debris in the drilling fluid, Ref. [11] proposed the improvement of ditch magnets through a combination of strong magnets and flow directors that would remove metallic swarf from the drilling fluid. These pre- or post-processing methods can effectively suppress well-known and constant magnetic interference sources of the drilling equipment; however, random and unknown magnetic interference (e.g., ore deposits) persist in causing poor azimuth measurement accuracy.

Software filtering methods play important roles in mitigating magnetic interference during the drilling operations. The typical rolling-based and digital filter-based (i.e., average filter, FIR, etc.) auto-calibration methods of magnetic interference are simple but are inapplicable to complex drilling environments [12]. To reduce the effects of perturbing magnetic fields associated with magnetized sections of the drill collar, Ref. [13] proposed a constant convergence algorithm that can iteratively estimate magnetic interference but which can only estimate axial constant errors. To eliminate the adverse effects azimuth that are imposed by accidental magnetic interference, Ref. [14] studied a new geomagnetic azimuth solution that involves integration of the magnetic and gravity readings. Given that the scalar product of two constant vectors will be constant in any coordinate system, Ref. [15] proposed a scalar product constant SPC-based magnetometer error calculation method on the prerequisite of an error-free accelerometer triad. The above software correction methods worked well, and with less severe magnetic interference, due to the poor immunity of the magnetometer-based MWD system to strong magnetic interference.

Recently, multi-azimuth fusion techniques, based on different sensory sources and free from magnetic interference, have been utilized to aid magnetometer-based MWD systems in resisting strong magnetic interference. To achieve high precision azimuth measurement, Ref. [2] used a Kalman filter (KF) to combine the geomagnetic azimuth and the azimuth derived from drilling trajectory prediction. Ref. [16] established the nonlinear model of orientation in a quaternion form, employing an unscented Kalman filter (UKF) to combine the geomagnetic azimuth solution and the azimuth calculated from a single gyroscope. Our prior work [17] studied the idea of extracting the geomagnetic vector in advance, before which the extracted geomagnetic information is robustly fused together with the azimuth derived from the gyroscope triad. Ref. [18] established a cut-off frequency-based error model of geomagnetic vector, and the azimuth was estimated through the fusion of triaxial gyroscopes and magnetometers. These studies aim to introduce aided azimuth sources, free of magnetic interference, into the magnetometer-based MWD system in order to achieve a high-precision azimuth measurement. However, both the trajectory prediction-based azimuth and integral-based azimuth by gyroscope triad are corrupted by errors that are accumulated over time due to the recursive strategy.

To resolve all of the aforementioned problems, this paper aims to develop a novel rotary norm constraint filtering (RNCF) method for azimuth estimation based on a gyroscope-aided magnetometer-based MWD system. This RNCF method features the use of KF to fuse geomagnetic measurement of the magnetometer triad and the geomagnetic state propagated by the Coriolis equation, based on the angular velocity measurement of the gyroscope triad of the BHA. In addition, given that the norm of the non-interfered geomagnetic vector can be approximated as a constant during a short-term drilling process, a norm constraint procedure is then introduced into the measurement update of the KF in order to further suppress magnetic interference by normalization of the interfered geomagnetic subset of the state vector. The geomagnetic estimate is then utilized to deliver the azimuth of the BHA. This azimuth estimation approach takes advantage of not incurring accumulative errors over time, as opposed to the multi-azimuth fusion approaches presented above.

The remainder of the paper is structured as follows: Section 2 shows the principle of the azimuth solution of the gyroscope-aided magnetometer-based MWD system. Section 3 describes the magnetic dynamical system model. A detailed RNCF method with which to obtain the desired geomagnetic vector is shown in Section 4. Section 5 presents experimental results and discussions. Section 6 concludes the results of the study.

## 2. Principle of the Azimuth Solution of a Gyroscope-Aided Magnetometer-Based MWD System

### 2.1. The Structure of the Gyroscope-Aided Magnetometer-Based MWD System

**Figure 1:**
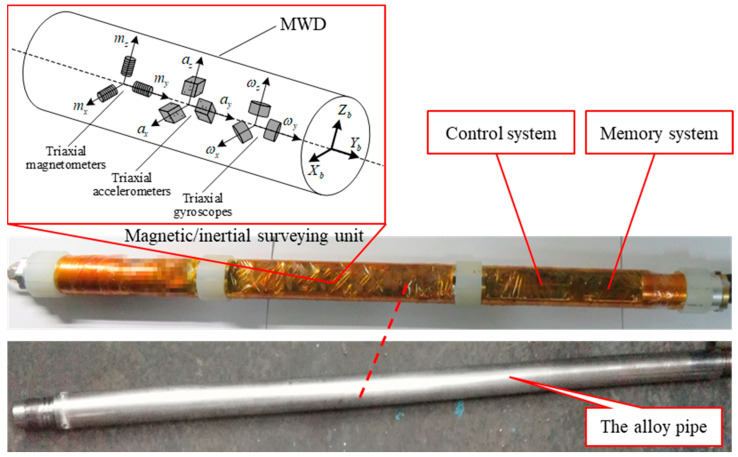
Gyroscope-aided magnetometer-based MWD system.

The gyroscope-aided magnetometer-based MWD system, introduced in this paper, consists of three parts: the control system, the memory system, and the magnetic/inertial surveying unit, as shown in Figure 1. The magnetic/inertial surveying unit incorporates anisotropic magnetoresistive triaxial magnetometers, micromechanical triaxial accelerometers and micromechanical triaxial gyroscopes. These are mounted in a titanium alloy pipe in three mutually orthogonal directions in order to measure the Earth’s magnetic field vector, the Earth’s gravity field vector, and the angular velocity vector in the BHA–body coordinate frame (b-frame), respectively. According to the harsh requirements of the drilling environment, the final candidates and the main performance parameters of the above magnetic and inertial sensors are listed in Table 1.

### 2.2. Principle of the Typical Geomagnetic Azimuth Solution of a Magnetometer-Based MWD System

**Figure 2:**
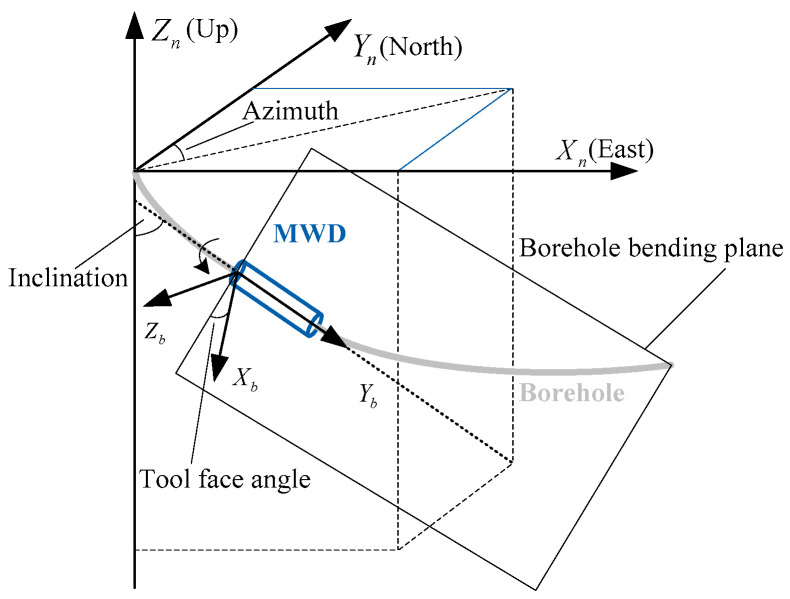
Definition of the orientation in geographic frame.

A geographic coordinate system is selected as the reference frame (n-frame) with which to represent the orientation (described by Euler angles, including inclination (θ), tool face angle (γ) and azimuth (ψ)) of the BHA, with the $X_{n}$, $Y_{n}$ and $Z_{n}$ axis aligned with the topographic north, east, and up directions, respectively, as shown in Figure 2.

Vector transformation from the b-frame to the n-frame can be described by the direction cosine matrix (DCM) $C_{b}^{n}$ in Euler angles form [19].

(1) $$C_{b}^{n}=\left[\begin{array}{ccc} \cos\psi \cos\gamma +\sin\psi \sin\theta \sin\gamma & -\cos\gamma \sin\psi +\sin\gamma \cos\psi \sin\theta & -\sin\gamma \cos\theta \\ \sin\psi \cos\theta & \cos\psi \cos\theta & \sin\theta \\ \sin\gamma \cos\psi -\cos\gamma \sin\psi \sin\theta & -\sin\gamma \sin\psi -\cos\psi \cos\gamma \sin\theta & \cos\gamma \cos\theta \end{array}\right]$$

At a certain orientation, triaxial accelerometers measure the gravitational field about the $X_{b}$, $Y_{b}$ and $Z_{b}$ axes of the BHA in the b-frame, termed $g_{x}^{b}$, $g_{y}^{b}$, and $g_{z}^{b}$, respectively. The outputs of the triaxial magnetometers, denoted by $m_{x}^{b}$, $m_{y}^{b}$ and $m_{z}^{b}$, represent the components of the Earth’s magnetic field in the b-frame. In particular, the gravitational field and geomagnetic vector in the n-frame can be taken as approximate constant vectors during the short-term drilling process, described as $g^{n}={\left[00g\right]}^{T}$ and $m^{n}={\left[0m\cos\beta -m\sin\beta \right]}^{T}$, where g is the magnitude of the local gravity acceleration, m is the local geomagnetic field strength, and β represents the local geomagnetic inclination.

The gravitational vector with regard to the b-frame ($g^{b}$) can be transformed from the n-frame ($g^{n}$) by $C_{n}^{b}$ as follows:

(2) $$g^{b}={\left[g_{x}^{b},g_{y}^{b},g_{z}^{b}\right]}^{T}=C_{n}^{b}{\left[0,0,g\right]}^{T},$$

where $C_{n}^{b}={\left(C_{b}^{n}\right)}^{T}$ is the DCM from the n-frame to the b-frame.

Similarly, the Earth’s magnetic vector of the b-frame ($m^{b}$) can be described as follows:

(3) $$m^{b}={\left[m_{x}^{b},m_{y}^{b},m_{z}^{b}\right]}^{T}=C_{n}^{b}{\left[0,mcos\beta ,-msin\beta \right]}^{T},$$

Combining (1), (2) and (3), the typical geomagnetic azimuth solution of BHA is given by the following:

(4) $$\psi =\arctan\frac{g(g_{x}^{b}m_{z}^{b}-m_{x}^{b}g_{z}^{b})}{m_{y}^{b}[{\left(g_{x}^{b}\right)}^{2}+{\left(g_{z}^{b}\right)}^{2}]-g_{y}^{b}(m_{x}^{b}g_{x}^{b}+m_{z}^{b}g_{z}^{b})}\pm D,$$

where $g_{x}^{b}$, $g_{y}^{b}$, $g_{z}^{b}$, $m_{x}^{b}$, $m_{y}^{b}$ and $m_{z}^{b}$ correspond respectively to gravity readings from the accelerometer triad and with magnetic readings from the magnetometer triad, while *D* defines the local geomagnetic declination, which can be premeasured.

### 2.3. Principle of the Azimuth Solution of a Gyroscope-Aided Magnetometer-Based MWD System

As can be readily seen from (4), the typical geomagnetic azimuth solution depends heavily on the magnetic outputs of the magnetometer triad. Unfortunately, magnetic interference may easily mask the desired azimuth-dependent component of the magnetic readings from the magnetometers, i.e., the geomagnetic vector, which leads to serious distortion of the azimuth during the drilling process. 

**Figure 3:**
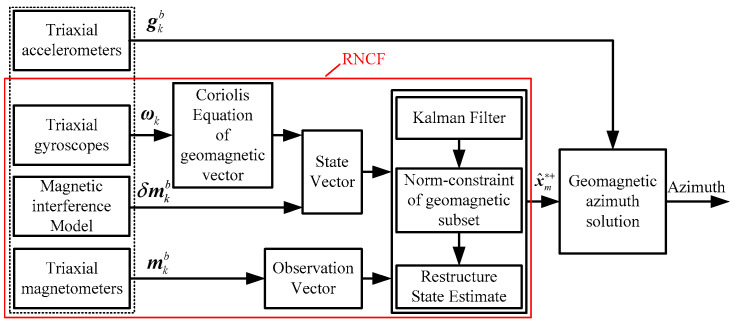
Workflow of the azimuth solution of a gyroscope-aided magnetometer-based MWD system.

For an accurate azimuth of the BHA, in this paper, a gyroscope triad is used to aid the typical magnetometer-based MWD system in shielding the typical geomagnetic azimuth solution from magnetic interference. The main workflow of the azimuth solution of a gyroscope-aided magnetometer-based MWD system is shown in Figure 3. A novel rotary norm constraint filtering (RNCF) method is designed to decouple the desired geomagnetic vector (${\hat{x}}_{m}^{*+}$) from the magnetic interference. Then, together with the Earth’s gravity vector from the accelerometer triad, the desired geomagnetic vector is used to deliver the azimuth of the BHA by (4).

## 3. Dynamical System Model of Geomagnetic Vector

As shown in Figure 3, the state vector of the proposed RNCF consists of two types of components of geomagnetic vector. One of these is the desired magnetic vector in the body frame of the BHA (termed as vector $m_{k}^{b}$), whose state can be propagated by the Coriolis equation using the angular velocity of the BHA [18]. This propagation process is described by the following:

(5) mkb=exp(−[ωk−1b×]⋅Ts)mk−1b=Fk−1mmk−1b,

where $\omega_{k-1}^{b}$ is the angular velocity vector of the BHA measured from the triaxial gyroscopes and $\left[\omega_{k-1}^{b}\times \right]$ is the skew symmetric matrix of $\omega_{k-1}^{b}$. Measurement noise of the gyroscopes leads to the propagation error of (5), which is given by the following:

(6) δm′kb≈−Ts[mk−1b×]wk−1g=Gk−1mwk−1g,

where $\delta {m^{\prime }}_{k}^{b}$ represents the propagation error, $\left[m_{k-1}^{b}\times \right]$ is the skew symmetric matrix of $m_{k-1}^{b}$, and wk−1g is the measurement noise of the gyroscopes at time *k* − 1.

The other component of the state vector is the external magnetic interference (termed vector $\delta m_{k}^{b}$), which can be approximatively characterized by a first-order Gaussian Markov (GM) process [18], the model of the magnetic interference is as follows:

(7) δmkb=k1⋅I3δmk−1b+k2⋅I3wk−1δ=Fδδmk−1b+Gδwk−1δ,

where $k_{1}$ is the correlation coefficient of the GM (the larger the $k_{1}$, the lower the frequency of the magnetic interference), $k_{2}$ is the random intensity of the GM, wk−1δ is assumed to be white Gaussian stimulation noise of the GM at *k* − 1 with zero mean and covariance matrix Rδ, $k_{1}$ and $k_{2}$ are all environment-dependent parameters, and $I_{3}$ is a 3 × 3 identity matrix.

To obtain the dynamical system model of geomagnetic vector, the two components of geomagnetic vector ($m_{k}^{b}$ and $\delta m_{k}^{b}$) are incorporated into the state of the dynamical system. The system’s state equation is given by the following:

(8) $$x_{k}=F_{k-1}x_{k-1}+G_{k-1}w_{k-1},$$

where $x_{k}={\left[m_{k}^{b},\delta m_{k}^{b}\right]}^{T}$ is the state vector, Fk−1=Fk−1m0303Fδ defines the transition matrix from time *k* − 1 to time *k*, Gk−1=Gk−1m0303Gδ is the noise coefficient matrix, wk−1=[wk−1g,wk−1δ]T is the noise vector of the state equation, and Gk−1m,Gδ, wk−1g and wk−1δ are the same as those presented in (6) and (7), respectively.

The observation vector of the dynamical system at time k denotes ykm, which can be measured from the triaxial magnetometers, which in turn can be described by the following:

(9) ykm=mkb+δmkb+wkm,

where wkm is the measurement noise of the triaxial magnetometers with zero mean and covariance matrix Rm.

The measurement equation of the dynamical system is then specified by the following:

(10) $$y_{k}=H_{k}x_{k}+v_{k},$$

where $H_{k}=[I_{3}I_{3}]$ describes the measurement matrix and vk=wkm is the measurement error vector.

## 4. Rotary Norm Constraint Filter Design

### 4.1. Kalman Filter Design

In this section, KF will be introduced to estimate the azimuth-dependent component $m_{k}^{b}$ based on the dynamical system model designed in Section 3.

The following time update equations are used to propagate the state estimate and covariance from one measurement time to the next, as follows:

(11) $$\left\{\begin{array}{l} {\hat{x}}_{k}^{-}=F_{k-1}{\hat{x}}_{k-1}^{+}\\ P_{k}^{-}=F_{k-1}P_{k-1}^{+}F_{k-1}^{T}+Q_{k-1} \end{array}\right.,$$

where ${\hat{x}}_{k}^{-}$ is the prior estimate of the state vector at time *k*, $F_{k-1}$ defines the transition matrix from time *k* − 1 to time *k*, ${\hat{x}}_{k-1}^{+}$ is the posteriori estimate of the state vector at time *k* − 1, $P_{k}^{-}$ describes the a priori covariance of the state vector at time *k*, $P_{k-1}^{+}$ is the a posteriori covariance of the state vector at time *k* − 1, and Qk−1=Gk−1RgI3I3RδGk−1T is the process noise covariance. 

The measurement update equations are given by the following:

(12) $$\left\{\begin{array}{l} K_{k}=P_{k}^{-}H_{k}^{T}{\left(H_{k}P_{k}^{-}H_{k}^{T}+R_{k}\right)}^{-1}\\ {\hat{x}}_{k}^{+}={\hat{x}}_{k}^{-}+K_{k}\varepsilon_{k}\\ P_{k}^{+}=(I-K_{k}H_{k})P_{k}^{-}{\left(I-K_{k}H_{k}\right)}^{T}+K_{k}R_{k}K_{k}^{T} \end{array}\right.,$$

where $K_{k}$ is the Kalman gain matrix at time *k*, $\varepsilon_{k}=y_{k}-H_{k}{\hat{x}}_{k}^{-}$ stands for the innovations, and $P_{k}^{+}$ is the a posteriori covariance of the state vector at time k.

### 4.2. Rotary Norm Constraint Filtering Method

It is noticeable that the norm of the geomagnetic subset ($m_{k}^{b}$) of the a posteriori state estimate (${\hat{x}}_{k}^{+}$) in (12) ideally equals the local geomagnetic intensity, while this condition is no longer true when there exists magnetic interference, especially strong magnetic interference. Thus, it is essential to add a normalization procedure of the geomagnetic part of the state estimate by minimizing a constrained cost function to guarantee a more precise geomagnetic subset estimate in KF. 

In this section, a novel rotary norm constraint filtering method corresponding with the above procedure will be introduced in order to improve the estimation accuracy of the geomagnetic vector based on the above KF design. For a clear understanding of the following derivation, we first define some notations that are used later. The subscript “*m*” denotes the variables associated with $m_{k}^{b}$, while the subscript “δ” denotes the variables related to $\delta m_{k}^{b}$. In addition, the superscript “*” is added to some variables in order to distinguish them from those of the KF.

Suppose that the 6 × 1 state vector $x_{k}$ is partitioned into $x_{m,k}$ and $x_{\delta ,k}$ as follows:

(13) $$x_{k}={\left[x_{m,k},x_{\delta ,k}\right]}^{T},$$

where $x_{m,k}$ stands for the desired Earth’s magnetic vector ($m_{k}^{b}$) and $x_{\delta ,k}$ denotes the magnetic interference ($\delta m_{k}^{b}$). The estimate error associated with each partition of $x_{k}$ will be minimized independently.

The estimate error covariance before the measurement update can be partitioned is as follows:

(14) $$P_{k}^{-}=\left[\begin{array}{cc} P_{m,k}^{-} & P_{\delta ,k}^{-} \end{array}\right]=\left[\begin{array}{c} {\left(P_{m,k}^{-}\right)}^{T}\\ {\left(P_{\delta ,k}^{-}\right)}^{T} \end{array}\right]=\left[\begin{array}{cc} P_{mm,k}^{-} & P_{m\delta ,k}^{-}\\ P_{\delta m,k}^{-} & P_{\delta \delta ,k}^{-} \end{array}\right],$$

The a posteriori covariance of the state vector is partitioned as follows:

(15) $$P_{k}^{+}=\left[\begin{array}{cc} P_{m,k}^{+} & P_{\delta ,k}^{+} \end{array}\right]=\left[\begin{array}{c} {\left(P_{m,k}^{+}\right)}^{T}\\ {\left(P_{\delta ,k}^{+}\right)}^{T} \end{array}\right]=\left[\begin{array}{cc} P_{mm,k}^{+} & P_{m\delta ,k}^{+}\\ P_{\delta m,k}^{+} & P_{\delta \delta ,k}^{+} \end{array}\right],$$

The Kalman gain is partitioned appropriately as follows:

(16) $$K_{k}={\left[K_{m,k},K_{\delta ,k}\right]}^{T},$$

To conduct the above procedure of the norm constraint of the state vector, $x_{m,k}$ is desired to have a predefined value, the constraint of which is equivalent to the following:

(17) $$\sqrt{{\left({\hat{x}}_{m,k}^{+}\right)}^{T}({\hat{x}}_{m,k}^{+})}=\left\|H\right\|,$$

where $\left\|H\right\|$ is the corrected norm of the Earth’s magnetic vector obtained by the IGRF model.

Combining (12) and (17), the state constraint can be expressed more conveniently as a constraint, as follows:

(18) $${\left({\hat{x}}_{m,k}^{-}+K_{m,k}\varepsilon_{k}\right)}^{T}({\hat{x}}_{m,k}^{-}+K_{m,k}\varepsilon_{k})={\left\|H\right\|}^{2},$$

Rearranging (18) yields the following:

(19) $$\varepsilon_{k}^{T}K_{m,k}^{T}K_{m,k}\varepsilon_{k}+2{\left({\hat{x}}_{m,k}^{-}\right)}^{T}K_{m,k}\varepsilon_{k}+{\left({\hat{x}}_{m,k}^{-}\right)}^{T}{\hat{x}}_{m,k}^{-}-{\left\|H\right\|}^{2}=0,$$

The a posteriori covariance matrix $P_{k}^{+}$, given by the Joseph formula of (12), can be rewritten as follows:

(20) $$P_{k}^{+}=P_{k}^{-}-K_{k}H_{k}P_{k}^{-}-P_{k}^{-}H_{k}^{T}K_{k}^{T}+K_{k}W_{k}K_{k}^{T},$$

where $W_{k}=H_{k}P_{k}^{-}H_{k}^{T}+R_{k}$.

Substituting (14), (15), and (16) into (20) and rearranging yields the following:

(21) $$P_{mm,k}^{+}=P_{mm,k}^{-}-K_{m,k}H_{k}P_{m,k}^{-}-{\left(P_{m,k}^{-}\right)}^{T}H_{k}^{T}K_{m,k}^{T}+K_{m,k}W_{k}K_{m,k}^{T},$$

(22) $$P_{\delta \delta ,k}^{+}=P_{\delta \delta ,k}^{-}-K_{\delta ,k}H_{k}P_{\delta ,k}^{-}-{\left(P_{\delta ,k}^{-}\right)}^{T}H_{k}^{T}K_{\delta ,k}^{T}+K_{\delta ,k}W_{k}K_{\delta ,k}^{T},$$

Equations (21) and (22) indicate that the matrix $P_{mm,k}^{+}$ is only a function of $K_{m,k}$, and that $P_{\delta \delta ,k}^{+}$ is only a function of $K_{\delta ,k}$. Additionally, the trace of $P_{k}^{+}$ is equal to the sum of the traces of $P_{mm,k}^{+}$ and $P_{\delta \delta ,k}^{+}$. The two facts imply that the minimum of the sum is equal to the sum of the minima, or that

(23) $$\underset{K_{k}}{\min}\left(tr(P_{k}^{+})\right)=\underset{K_{k}}{\min}\left(tr(P_{mm,k}^{+})+tr(P_{\delta \delta ,k}^{+})\right)=\underset{K_{m,k}}{\min}\left(tr(P_{mm,k}^{+})\right)+\underset{K_{\delta ,k}}{\min}\left(tr(P_{\delta \delta ,k}^{+})\right)$$

Equation (23) suggests that the optimal gain $K_{k}$ can be calculated independently by minimizations of the two portions of $K_{m,k}$ and $K_{\delta ,k}$.

Calculating $\partial [tr(P_{\delta \delta ,k}^{+})]/\partial K_{\delta ,k}=0$ yields the following:

(24) $$K_{\delta ,k}={\left(P_{\delta ,k}^{-}\right)}^{T}H_{k}^{T}W_{\delta ,k}^{-1},$$

where $\partial [tr(P_{\delta \delta ,k}^{+})]/\partial K_{\delta ,k}$ represents the partial derivative of the trace of $P_{\delta \delta ,k}^{+}$ to $K_{\delta ,k}$.

Similarly, according to $\partial [tr(P_{mm,k}^{+})]/\partial K_{m,k}=0$, the optimal gain of $x_{m,k}$ is:

(25) $$K_{m,k}={\left(P_{m,k}^{-}\right)}^{T}H_{k}^{T}W_{k}^{-1},$$

The a posteriori estimate (KF) of $x_{m,k}$ is as follows:

(26) $${\hat{x}}_{m,k}^{+}={\hat{x}}_{m,k}^{-}+{\left(P_{m,k}^{-}\right)}^{T}H_{k}^{T}W_{k}^{-1}\varepsilon_{k},$$

The Kalman gain is recomputed in order to satisfy the constraint in (19), and the augmented performance index of $K_{m,k}$ is given by the following:

(27) $$J=tr(P_{mm,k}^{+})+\lambda_{k}\left(\varepsilon_{k}^{T}K_{m,k}^{T}K_{m,k}\varepsilon_{k}+2{\left({\hat{x}}_{m,k}^{-}\right)}^{T}K_{m,k}\varepsilon_{k}+{\left({\hat{x}}_{m,k}^{-}\right)}^{T}{\hat{x}}_{m,k}^{-}-{\left\|H\right\|}^{2}\right),$$

where $\lambda_{k}$ is the Lagrange multiplier.

The performance index “J” is minimized and the constraint is simultaneously satisfied when the optimal gain is chosen as follows:

(28) $$K_{m,k}^{*}=K_{m,k}+(\frac{\left\|H\right\|}{\left\|{\hat{x}}_{m,k}^{+}\right\|}-1){\hat{x}}_{m,k}^{+}\frac{\varepsilon_{k}^{T}W_{k}^{-1}}{\varepsilon_{k}^{T}W_{k}^{-1}\varepsilon_{k}},$$

where $K_{m,k}^{*}$ defines the constrained Kalman gain of $x_{m,k}$. Further details related to solving the constrained optimization of (28) are listed in the Appendix A. 

The measurement update of $x_{m,k}$ after the norm constraint of the desired geomagnetic vector is given by the following:

(29) $${\hat{x}}_{m,k}^{*+}={\hat{x}}_{m,k}^{-}+K_{m,k}^{*}\varepsilon_{k},$$

Combining (25), (26), (28) and (29) and rearranging yields the following:

(30) $${\hat{x}}_{m,k}^{*+}=\frac{\left\|H\right\|}{\left\|{\hat{x}}_{m,k}^{+}\right\|}{\hat{x}}_{m,k}^{+},$$

Substituting $K_{m,k}^{*}$ into (21) and rearranging yields the a posteriori covariance matrix of ${\hat{x}}_{m,k}^{*+}$:

(31) $$P_{mm,k}^{*+}=(I-K_{m,k}^{*}H_{k})P_{m,k}^{-}{\left(I-K_{m,k}^{*}H_{k}\right)}^{T}+K_{m,k}^{*}R_{k}{\left(K_{m,k}^{*}\right)}^{T},$$

Therefore, (28), (30), and (31) are the measurement update operations in regard to norm constraint.

## 5. Experimental Results

### 5.1. Simulation Experiments

In this experiment, a simulation is first conducted in order to validate the proposed RNCF method by MATLAB (2018a), and the motion trajectory of the gyroscope-aided magnetometer-based MWD system is designed. The gyroscope-aided magnetometer-based MWD system turns around the Zn-axis in circles on a horizontal plane, while the theoretical parameters of the azimuth motion trajectory, including triaxial angular velocity, triaxial geomagnetic vector (or the projection of the geomagnetic vector on the horizontal plane), and the azimuth of the MWD, are generated by a generator. Given this, the raw triaxial angular velocity data are obtained from a mixture of theoretical data and noise based on the selected gyroscope listed in Section 2. Meanwhile, for a more realistic simulation of the strong magnetic disturbance in the drilling process, the raw triaxial magnetic field data are obtained from the theoretical data with the addition of substantial random magnetic disturbance noise and measurement noise derived from the magnetometers.

The parameters of the generated azimuth motion trajectory are as follows: first, the geomagnetic field strength is 0.5257 Gauss, the magnetic declination is 4.93° (W), and the inclination is 53.77°. These parameter settings are geographically related to the laboratory. Second, the random magnetic disturbance is modeled as a first-order GM process of (7) with $k_{1}=0.9$ and $k_{2}=0.02$. Third, the theoretical triaxial angular velocity is (0;0;10°), the original azimuth is 355.07° and the sampling point N is 7200 with the sampling 50 Hz.

**Figure 4:**
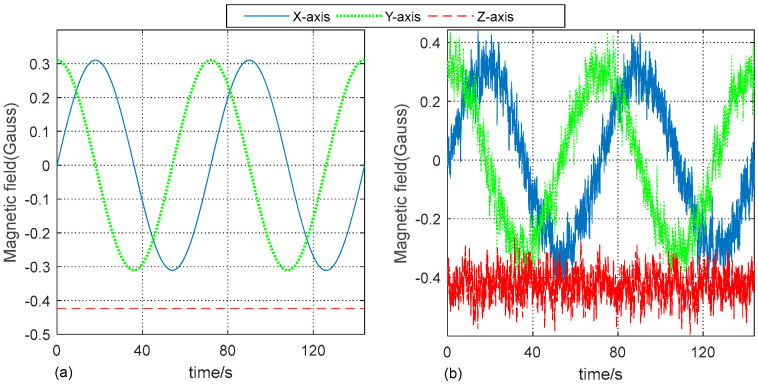
The triaxial magnetic data of the simulation test setup. (**a**) The theoretical data and (**b**) the raw triaxial magnetic data in the simulation.

The simulation experiments are conducted as follows: first, the theoretical and raw data are generated, as shown in Figure 4. Second, the raw magnetic data are filtered by the proposed RNCF method, compared with the typical geomagnetic filter [13] and KF [2] widely utilized in MWD. Third, the root mean square error (RMSE), norm and azimuth of these magnetic data are calculated in order to evaluate filtering performance.

The main parameters of the KF and the proposed RNCF are set as follows: Rg=0.0004, Rm=0.0001, $k_{1}=0.9$, $k_{2}=0.02$, and $\left\|H\right\|=0.5257$. The initial state vector and the initial covariance are as follows: $x_{0}={\left[0,0.5257,0,0.01,0.01,0.01\right]}^{T}$, $P_{0}=diag\left\{0.1,0.1,0.1,0.2,0.2,0.2\right\}$, where the sensor-related parameters are set based on Table 1.

**Figure 5:**
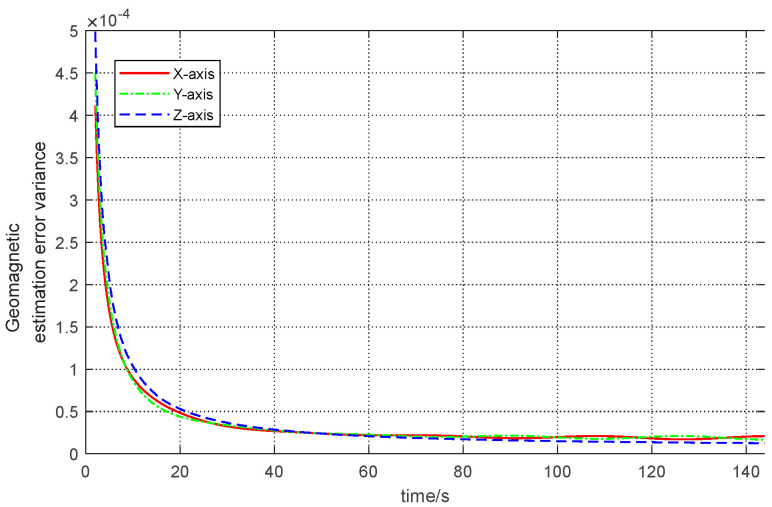
The a posteriori geomagnetic estimation error variance of the proposed RNCF.

**Figure 6:**
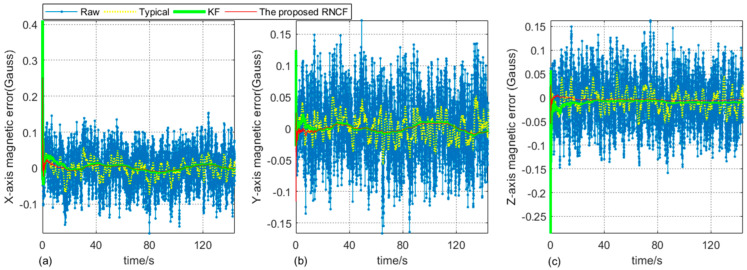
Triaxial magnetic errors of the raw, typical geomagnetic filter, KF, and the proposed RNCF, respectively. (**a**) X-axis. (**b**) Y-axis. (**c**) *Z*-axis.

**Figure 7:**
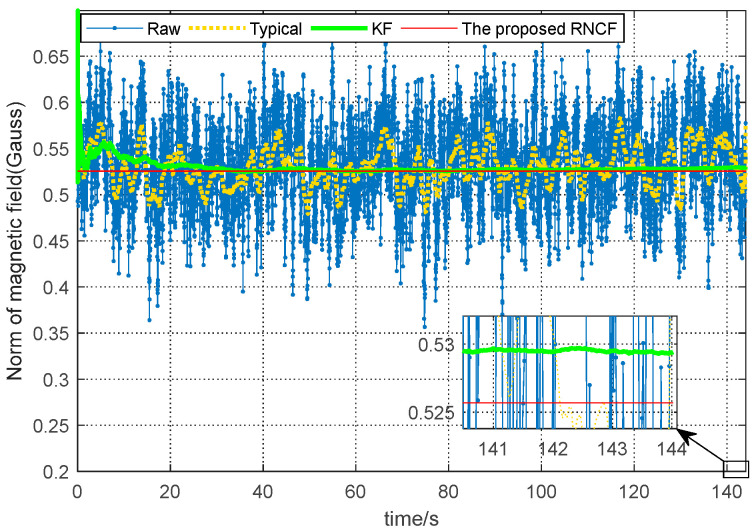
Comparison of the norm of the magnetic data.

**Figure 8:**
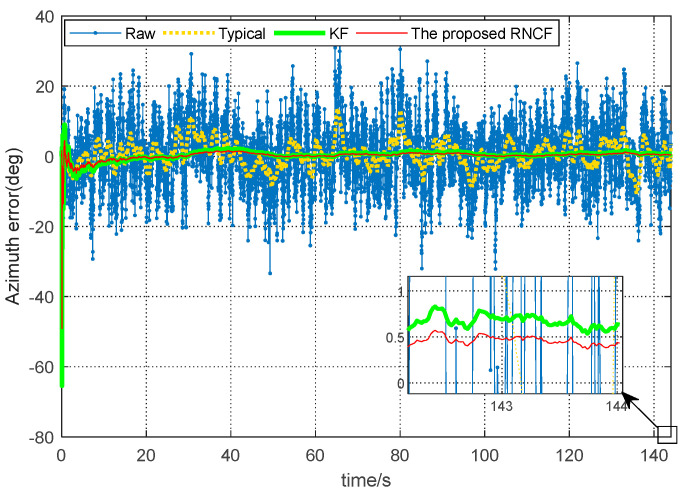
Comparison of the azimuth error of the magnetic data.

Then, the simulation experiments are repeated five times with each using a different length of the 7200 data to reduce accidental errors. A certain trial is shown in Figure 4, Figure 5, Figure 6, Figure 7 and Figure 8. The results of the RMSE values are summarized in Table 2, where each value stands for the mean of all five experiments.

At first, the stability and reliability of the proposed RNCF is validated by the variance of the geomagnetic estimate ($P_{mm}^{-},P_{mm}^{+}$) of the RNCF, as shown in Figure 5. It can be seen from this figure that the variance converges to a steady-state value, which indicates that the proposed RNCF is stably convergent.

As can be seen from Figure 6, some random magnetic disturbance in raw triaxial magnetic data have been removed by the typical geomagnetic filter, while there is substantial random magnetic disturbance by KF and the proposed RNCF. In addition, Table 2 further shows that the filtering performance of the proposed RNCF is superior to KF or to the typical geomagnetic filter in a RMSE sense.

As shown in Figure 7, the norm of the raw triaxial magnetic data shows substantial fluctuations between 0.35 and 0.70 Gauss with an RMSE 0.0475 Gauss. Due to the strong magnetic disturbance, the norm of the typical geomagnetic filter presents some between 0.47 and 0.58 Gauss with an RMSE 0.0215 Gauss. The norm of the KF shows some fluctuations between 0.51 and 0.69 Gauss with an RMSE 0.0082 Gauss, while the proposed RNCF always maintains 0.0057 Gauss (the geomagnetic field strength) due to the normalization of the geomagnetic subset of the state vector.

Figure 8 shows the azimuth errors determined from the raw magnetic readings, the typical geomagnetic solution, the KF, and the proposed RNCF, followed by the steady-state azimuth error values (the last length—500) in Table 3. As detailed in Figure 8 and Table 3, the azimuth error determined from the raw magnetic data shows great fluctuations between −33.4° and 36.6°, the typical geomagnetic azimuth solution fluctuates between −10.6° and 13.5°, while the steady-state errors of the azimuth derived from KF and the proposed RNCF are relatively smaller, within 1°, while the steady-state error of the proposed RNCF is limited to within 0.6° and outperforms the KF within 1°. It can be concluded that the performance improvements of the proposed RNCF over the typical geomagnetic solution and KF are, respectively, 98.5% and 37.1% for the azimuth measurement precision in an RMSE sense, which suggests that the proposed RNCF achieves the highest azimuth measurement precision compared with the typical and KF. However, the performance of the proposed RNCF depends on the rotary angular velocity of the BHA. The higher the rotating speed of the BHA, the better the observability of the change rate of the geomagnetic vector sensed by angular velocity, and the higher the accuracy of the proposed RNCF.

### 5.2. Actual Drilling Experiment

To further demonstrate the feasibility of the proposed RNCF method to strong magnetic interference, the real field-test data are selected for testing. Meanwhile, the azimuth derived from the triaxial magnetic readings using the proposed RNCF method is compared with the typical geomagnetic solution [10] and the triaxial magnetic readings using KF [2]. 

**Figure 9:**
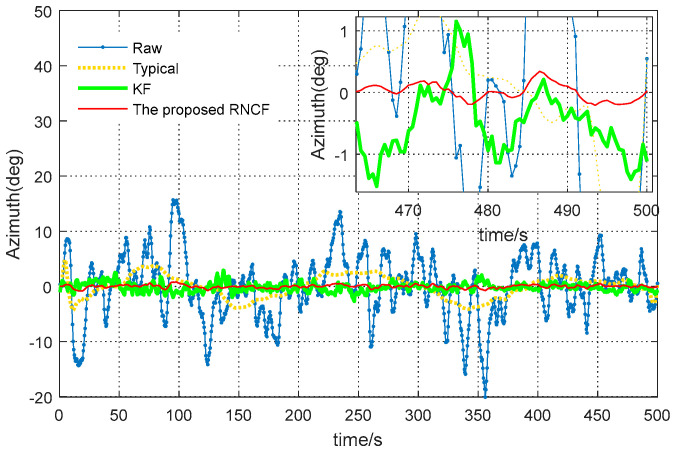
Comparison of the azimuths from raw magnetic readings, the typical geomagnetic solution, KF, and the proposed RNCF.

**Figure 10:**
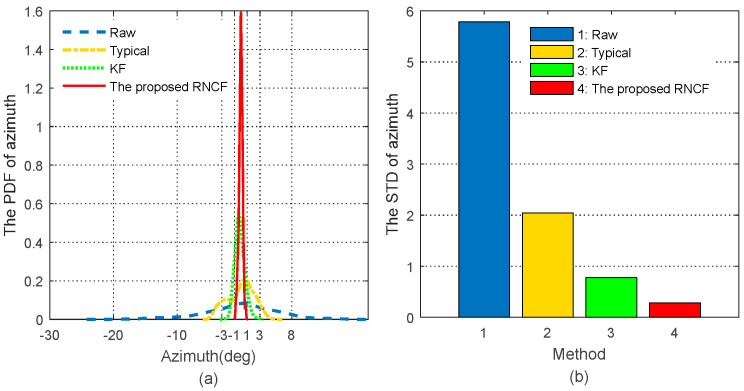
Comparison of the statistical parameters of the azimuths from raw magnetic readings, the typical geomagnetic solution, KF, and the proposed RNCF. (**a**) The PDF of the azimuth. (**b**) The STD of the azimuth.

In the actual continuous drilling process, there is no method that could be used to obtain the real azimuth of the BHA, when failing to accurately evaluate the accuracy of the azimuth of the BHA. In this paper, the stability of the azimuth of the BHA during a horizontal directional drilling process with a small fluctuation in azimuth will be used to assess the accuracy [2]. Experimental data have been specially collected from the MWD surveying system shown in Section 2 in a stationary horizontal drilling process with substantial magnetic interference in the Tarim oilfield, Xinjiang, China, from June, 2017. The sampling frequency of the magnetic and inertial surveying unit is 50 Hz, while the output frequency of the off-line azimuth determined from the collected magnetic and inertial data by the above four methods is 0.5 Hz. For better observation of the stability of the azimuth, the relevant azimuth values are removed from their respective average value, and the final results are detailed in Figure 9, followed by the probability density function (PDF) and standard deviation (STD) of those final azimuths, shown in Figure 10.

As evident in Figure 9, the azimuth derived from the raw magnetic readings and the typical geomagnetic solution show significant fluctuations. This is because of their poor immunity to the strong magnetic interference, while azimuth derived from the magnetic readings filtered by KF and RNCF are relatively smaller, due to the assistance of the gyroscope triad.

Figure 10 further shows the stability of these azimuths. The PDF of the raw magnetic readings is the widest and shortest one, with the greatest fluctuation between −20° and 16° and the highest STD of 5.7869, while the proposed RNCF is the narrowest and highest one, with the smallest fluctuation within ± 1° and with the shortest STD of 0.2832. The KF shows better stability within ± 3° with the STD of 0.7761, compared with the typical geomagnetic azimuth solution, which ranges from −10° to 8° with an STD of 2.7339. It is not hard to conclude that the stability improvement of the azimuth of the proposed RNCF over the typical geomagnetic solution is about 89.6% and the KF 63.5% in an STD sense, which indicates that the proposed RNCF method presents the best stability and determines the accurate azimuth of the BHA with the highest probability compared with the typical geomagnetic solution and the KF mentioned above. 

## 6. Conclusions

As presented, the typical geomagnetic azimuth solution of the magnetometer-based MWD system is susceptible to interference while drilling, especially strong magnetic interference. Aiming to address this problem, this paper manages to develop a novel rotary norm constraint filtering method (RNCF) for azimuth estimation in order to decouple the desired geomagnetic vector from magnetic interference by a gyroscope-aided magnetometer-based MWD system. The novelty of the RNCF method is that two features that are easily implemented in the drilling process are utilized. One of these is that the desired geomagnetic vector of the BHA can be equivalently estimated by the Coriolis equation using the angular velocity measurement of the gyroscope triad, and the other is the norm constraint of the desired geomagnetic vector. Experimental results show that the proposed RNCF method can effectively decouple the desired geomagnetic field from the magnetic interference and improve the azimuth measurement precision by 98.5% over the typical geomagnetic solution and 37.1% over KF in an RMSE sense in a strong magnetic interference environment.

Despite the performance enhancement in azimuth accuracy, the proposed method still has room for improvement. The proposed method mainly focuses on magnetic interference from the well-known interference sources that derive from drilling equipment and random sources from the nearby environment during the drilling process (e.g., the randomly located ore deposits). As a result, future work should consider the possible magnetic interference from the operation of the drilling process, e.g., rotation speed, which might contribute to the improvement of the accuracy performance of the azimuth.

## Figures and Tables

**Table 1:** Characteristics of the magnetic/inertial sensors.

Parameters	Magnetometer (HMC1043)	Accelerometer (MS9010)	Gyroscope (CRG20-02)
Range	±6 gauss	±10 g	±300°/s
Resolution	120 μgauss	0.1 mg	0.03125°/s
Bias stability	-	<0.25 mg	4.7°/h
Noise	50 nV/√Hz	0.140 mg/√Hz	18 nV/√Hz
Working TEMP	−40–125 °C	−55–125 °C	−40–105 °C

**Table 2:** Comparison of the RMSE of the different magnetic field data.

Magnetic Field (Gauss)	Raw	Typical	KF	The Proposed
RMSE (*X*-axis)	0.0472	0.0222	0.0121	0.0061
RMSE (*Y*-axis)	0.0466	0.0195	0.0090	0.0047
RMSE (*Z*-axis)	0.0470	0.0211	0.0129	0.0033

**Table 3:** Comparison of the azimuth error of the four methods.

Methods	Raw	Typical	KF	The Proposed
Maximum error (°)	36.62	13.50	0.96	0.63
Minimum error (°)	−33.41	−10.62	−0.93	−0.51
Mean error (°)	−1.59	−1.53	0.79	0.54
Steady-state RMSE (°)	52.17	38.64	0.89	0.56

## Data Availability

Data are contained within the article.

## Appendix A

The following derivations are used to obtain (28):

Calculating $\partial (J)/\partial K_{m,k}=0$ yields the following:

(A1) $$-2{\left(P_{m,k}^{-}\right)}^{T}H_{k}^{T}+2K_{m,k}W_{k}+2\lambda_{k}({\hat{x}}_{m,k}^{-}\varepsilon_{k}^{T}+K_{m,k}\varepsilon_{k}\varepsilon_{k}^{T})=0$$

The $K_{m,k}$ satisfied with (A1) is just the $K_{m,k}^{*}$. Using the matrix inversion lemma, it follows that

(A2) $$\begin{array}{l} K_{m,k}^{*}={\left(P_{m,k}^{-}\right)}^{T}H_{k}^{T}W_{k}^{-1}-\lambda_{k}{\hat{x}}_{m,k}^{-}\varepsilon_{k}^{T}W_{k}^{-1}-{\left(P_{m,k}^{-}\right)}^{T}H_{k}^{T}W_{k}^{-1}\frac{\lambda_{k}\varepsilon_{k}\varepsilon_{k}^{T}W_{k}^{-1}}{1+\lambda_{k}\varepsilon_{k}^{T}W_{k}^{-1}\varepsilon_{k}}\\ +\lambda_{k}{\hat{x}}_{m,k}^{-}\varepsilon_{k}^{T}W_{k}^{-1}\frac{\lambda_{k}\varepsilon_{k}\varepsilon_{k}^{T}W_{k}^{-1}}{1+\lambda_{k}\varepsilon_{k}^{T}W_{k}^{-1}\varepsilon_{k}} \end{array}$$

Substituting (A2) into (19) and rearranging yields the following:

(A3) $$\begin{array}{l} -\lambda_{k}^{2}{\left\|H\right\|}^{2}{\left(\varepsilon_{k}^{T}W_{k}^{-1}\varepsilon_{k}\right)}^{2}-\lambda_{k}\left(2{\left\|H\right\|}^{2}\varepsilon_{k}^{T}W_{k}^{-1}\varepsilon_{k}\right)+\varepsilon_{k}^{T}W_{k}^{-1}H_{k}P_{m,k}^{-}{\left(P_{m,k}^{-}\right)}^{T}H_{k}^{T}W_{k}^{-1}\varepsilon_{k}\\ +2{\left({\hat{x}}_{m,k}^{-}\right)}^{T}{\left(P_{m,k}^{-}\right)}^{T}H_{k}^{T}W_{k}^{-1}\varepsilon_{k}+{\left({\hat{x}}_{m,k}^{-}\right)}^{T}{\hat{x}}_{m,k}^{-}-{\left\|H\right\|}^{2}=0 \end{array}$$

Finally, it follows that

(A4) $$\lambda_{k}=\frac{-1}{\varepsilon_{k}^{T}W_{k}^{-1}\varepsilon_{k}}\pm \frac{\left\|{\hat{x}}_{m,k}^{-}+{\left(P_{m,k}^{-}\right)}^{T}H_{k}^{T}W_{k}^{-1}\varepsilon_{k}\right\|}{\varepsilon_{k}^{T}W_{k}^{-1}\varepsilon_{k}\left\|H\right\|}$$

Substituting (A4) into (A2) and combining (25) and (26) yields (28), as follows:

$$K_{m,k}^{*}=K_{m,k}+(\frac{\left\|H\right\|}{\left\|{\hat{x}}_{m,k}^{+}\right\|}-1){\hat{x}}_{m,k}^{+}\frac{\varepsilon_{k}^{T}W_{k}^{-1}}{\varepsilon_{k}^{T}W_{k}^{-1}\varepsilon_{k}}$$
